# A Novel Ferroptosis-Related Gene Risk Signature for Predicting Prognosis and Immunotherapy Response in Gastric Cancer

**DOI:** 10.1155/2021/2385406

**Published:** 2021-11-26

**Authors:** Shi-jin Liu, Ya-bing Yang, Jia-xin Zhou, Yu-jian Lin, Yun-long Pan, Jing-hua Pan

**Affiliations:** ^1^Department of General Surgery, The First Affiliated Hospital of Jinan University, Guangzhou 510632, China; ^2^International School, Jinan University, Guangzhou, Guangdong 510632, China; ^3^MOE Key Laboratory of Tumor Molecular Biology and Key Laboratory of Functional Protein Research of Guangdong Higher Education Institutes, Institute of Life and Health Engineering, Jinan University, Guangzhou 510632, China

## Abstract

**Background:**

Gastric cancer (GC) is the third leading cause of cancer death worldwide with complicated molecular and cellular heterogeneity. Iron metabolism and ferroptosis play crucial roles in the pathogenesis of GC. However, the prognostic role and immunotherapy biomarker potential of ferroptosis-related genes (FRGs) in GC still remains to be clarified.

**Methods:**

We comprehensively analyzed the prognosis of different expression FRGs, based on gastric carcinoma patients in the TCGA cohort. The functional enrichment and immune microenvironment associated with these genes in gastric cancer were investigated. The prognostic model was constructed to clarify the relation between FRGs and the prognosis of GC. Meanwhile, the ceRNA network of FRGs in the prognostic model was performed to explore the regulatory mechanisms.

**Results:**

Gastric carcinoma patients were classified into the A, B, and C FRGClusters with different features based on 19 prognostic ferroptosis-related differentially expressed genes in the TCGA database. To quantify the FRG characteristics of individual patients, FRGScore was constructed. And the research shows the GC patients with higher FRGScore had worse survival outcome. Moreover, thirteen prognostic ferroptosis-related differentially expressed genes (DEGs) were selected to construct a prognostic model for GC survival outcome with a superior accuracy in this research. And we also found that FRG RiskScore can be an independent biomarker for the prognosis of GC patients. Interestingly, GC patients with lower RiskScore had less immune dysfunction and were more likely to respond to immunotherapy according to TIDE value analysis. Finally, a ceRNA network based on FRGs in the prognostic model was analyzed to show the concrete regulation mechanisms.

**Conclusions:**

The ferroptosis-related gene risk signature has a superior potent in predicting GC prognosis and acts as the biomarkers for immunotherapy, which may provide a reference in clinic.

## 1. Introduction

Gastric cancer is the fifth of the most common cancers worldwide and the third of the most common causes of cancer death all around the world. With more than 1 million new cases estimated each year and often diagnosed at an advanced stage, gastric cancer owns a high mortality rate, with 784,000 deaths worldwide in 2018 [1]. With the progress in treatment and diagnosis, the clinical prognosis of GC patients has been significantly improved [2]. However, because of the lack of classical symptoms in early stage, the diagnosis of GC is challengeable, as clinical symptoms often appear at late stage during GC development, narrowing the options for GC treatment and showing the significance of biomarkers. Intratumoral, interpatient, and intrapatient heterogeneity in gastric cancer remains as a hard barrier to the prognosis of patients with GC. Therefore, more researches are needed to explore the underlying mechanism of GC [3]. And the study of the prognostic biomarkers and relevant predictive models in clinic becomes an urgent demand for GC diagnosis and therapy. What is more, with the development of high technology and the better knowledge about human body we learn, immunotherapy has become one of the most popular topics in cancer researches. Biomarkers are playing a more and more important role in GC prognosis and GC therapy, including TMB, IFNGR1, and TNFRSF19L. Nevertheless, the effective biomarkers are still short today and only a few conventional biomarkers can be used in clinic such as CEA and CA19-9. In addition, some existing biomarker still needs more researches to study concrete further mechanism. For example, the efficiency of predicting immunotherapy by TMB still remains controversial [4]. In this way, it is a great need to do relative studies and find out new biomarkers of immunotherapy for GC.

Ferroptosis is a form of regulated cell death, which was newly discovered and has become more and more popular in cancer research field because of the involvement in development, immunity, senescence, and a variety of pathological situations. Ferroptosis is defined as an oxidative, iron-dependent form of “regulated cell death” (RCD) which has the characters of reactive oxygen species (ROS) accumulation and lipid peroxidation product accumulation to lethal level [5, 6]. Nowadays, ferroptosis has received a lot of interests, especially in consideration of the downregulation and gene silencing involved in the initiation and execution of necroptosis of cancers. In spite of the important roles of ferroptosis in sustaining normal cells and tissue survival, it has been increasingly recognized that some oncogenic pathways are associated with ferroptosis, causing cancer cells extremely vulnerable to ferroptosis death [7]. Some studies have also confirmed the essential importance of ferroptosis for GC treatment and prognosis, such as the facilitation of perilipin2 in regulating the proliferation and apoptosis of gastric carcinoma cells by modification in the ferroptosis pathway [8]. On the other hand, CAF-secreted miR-522 suppresses ferroptosis and ultimately results in decreased chemosensitivity in gastric cancer [9]. However, whether these genes related to ferroptosis can be identified as effective potential diagnostic biomarkers and therapeutic targets to combat GC, thereby helping to improve the survival prognosis of patients with GC, needs further researches.

In this research, we comprehensively analyzed the different expressions and prognoses of FRGs in GC patients based on public databases and constructed a prognostic model based on thirteen FRGs. The different sensitivity of immunotherapy in gastric cancer patients provided potent clinical reference. Besides, ceRNA network has been proved playing an important role in cancer process. Loads of studies have demonstrated that long noncoding RNAs (lncRNAs) can bind to microRNA (miRNA) sites as competing endogenous RNAs (ceRNAs) to regulate the expression of mRNA and target genes [10]. We also analyzed the relationship between ferroptosis-related gene signatures and ceRNA network, and the circRNA-miRNA-lncRNA-mRNA network was created to reveal the mechanism of GC.

## 2. Materials and Methods

### 2.1. Acquisition of Gastric Cancer Datasets and Ferroptosis-Related Genes

The RNA-seq data and corresponding clinical information were acquired from the TCGA database (https://portal.gdc.cancer; including 375 GC samples and 32 normal tissue samples). And circRNA expression profile data in GC patients were downloaded from the GEO database (GSE174237, including 6 GC samples and 6 corresponding normal tissue samples). Ferroptosis-related genes were obtained from the FerrDb database (http://www.zhounan.org/ferrdb/index.html). These data are available in public, and the approval of the local ethics committee is not required. In the course of our research, we strictly abided by the rules for the use of these databases.

### 2.2. Identified DEGs Associated with GC Prognosis

Matching the mRNA-sequencing data with FRGs and the differentially expressed genes (DEGs) between GC tissues and adjacent nontumorous tissues was identified by the “limma” R package with a false discovery rate of *P* < 0.05. Univariate Cox analysis of overall survival (OS) was performed using the “survival” R package to screen FRGs with prognostic potential. An interaction network for the overlapping prognostic DEGs was generated by the STRING database (https://www.string-db.org/).

### 2.3. Consensus Clustering Analysis and Construction of FRGScore

The prognostic DEGs were incorporated to divide GC patients in the TCGA cohort into different clusters with “ConsensusClusterPlus” R package. Kaplan-Meier analysis was used to evaluate the differences of OS between different clusters. Thereafter, principal component analysis (PCA) was used to validate the reliability of clustering. We performed GSVA enrichment analysis to investigate the difference of biological process between different clusters. Then, we constructed a set of scoring system to evaluate the FRG pattern of individual GC patients based on principal component analysis, termed as FRGScore. Both principal components 1 and 2 were selected to act as scores. Time-dependent receiver operating characteristic (ROC) curve analysis was performed to evaluate the predictive power of FRGScore and other clinical factors. The infiltration of immune cells was assessed between patients with different FRGScores through the CIBERSORT computational method.

### 2.4. Correlation between FRGScore and Tumor Mutational Burden (TMB)

The somatic mutation data was acquired from the TCGA database. We analyzed the distribution differences of somatic mutation using the maftools package. A correlation analysis was performed then, to further reveal the association between FRGScore and tumor mutation.

### 2.5. Development of Prognostic Signatures Based on FRGs

With expression profiles of the identified survival-associated FRGs, least absolute shrinkage and selection operator (LASSO) regression analysis was conducted through the “glmnet” R package. The RiskScore of the FRG model for each patient was calculated as follows:

(1) $$\begin{array}{c} \text{RiskScore}=\overset{n}{\underset{i=1}{\sum }}\left(\text{Exp}i*\beta i\right), \end{array}$$

where *n* is the number of selected FRGs, Exp*i* is the expression value of gene *i*, and *βi* is the coefficient of gene *i* generated from LASSO regression analysis. To determine whether the RiskScore was an independent prognostic predictor for OS compared to other clinical features, univariate and multivariate Cox regression analyses were performed.

### 2.6. Immunotherapy Response Predictions

TIDE (http://tide.dfci.harvard.edu/) is a computational method which integrates the expression signatures of T cell dysfunction and exclusion to model tumor immune evasion. The TIDE algorithm was used to predict the clinical response to immune checkpoint blockade (ICB) in GC patients on the basis of pretreatment genomics.

### 2.7. Construction and Evaluation of the Nomogram

The “rms” R package was used to construct a predictive nomogram and corresponding calibration maps based on independent predictive factors. ROC curves were generated to determine the sensitivity and specificity of the predictive nomogram.

### 2.8. Construction of circRNA–miRNA–lncRNA–mRNA Network

GDCRNATools were performed to identify miRNAs targeting FRGs in prognostic models and lncRNAs based on differential lncRNAs and miRNAs between tumorous and nontumorous samples of GC patients in TCGA cohorts. Tumorous and nontumorous samples of GSE174237 were used to screen for circRNA with abnormal expression in tumors. The circRNA bound to miRNA was predicted using the starBase database (http://starbase.sysu.edu.cn/). Finally, the intersection of circRNA–miRNA, miRNA-lncRNA, and miRNA–mRNA pair was taken to construct the circRNA–miRNA–lncRNA–mRNA regulatory network.

### 2.9. Statistical Analysis

All statistical analyses were performed using the R software (version 4.0.3). Student's two-sided *t*-test was performed to compare gene expression between GC tissues and adjacent nontumorous tissues. The OS of different groups was compared by Kaplan-Meier analysis followed by the log-rank test. All *P* values were two-tailed. A *P* value < 0.05 was considered statistically significant if not specified above.

## 3. Results

### 3.1. Identification of Ferroptosis-Related Prognostic DEGs in the TCGA Database

**Figure 1:**
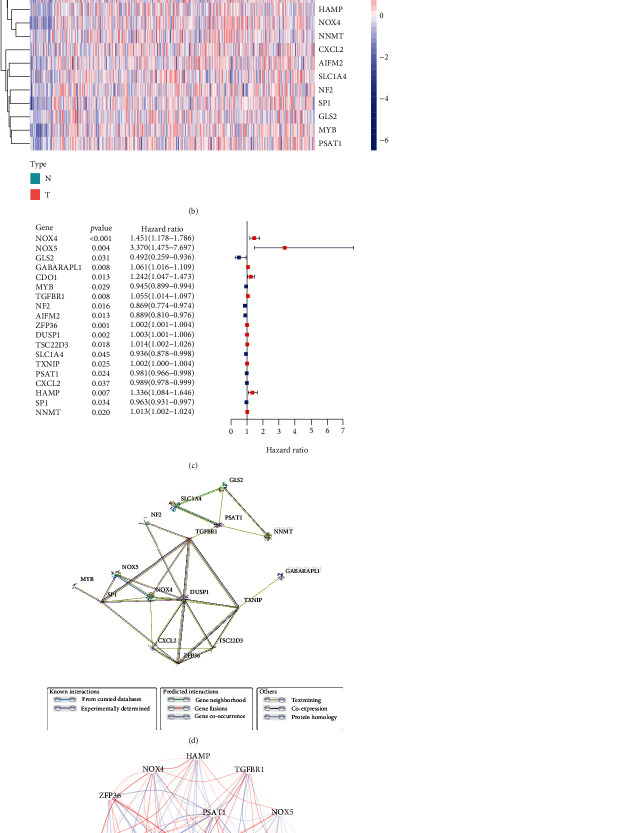
Identification of the candidate ferroptosis-related genes in the TCGA cohort. (a) Venn diagram to identify differentially expressed genes between tumor and adjacent normal tissue that were correlated with OS. (b) The expression of the 19 overlapping genes in normal and tumor tissues. (c) Forest plots showing the results of the univariate Cox regression analysis between gene expression and OS. (d) The PPI network downloaded from the STRING database indicated the interactions among the candidate genes. (e) The correlation network of candidate genes. The correlation coefficients are represented by different colors.

164 differential expression FRGs between GC tumor samples and adjacent normal samples and 27 OS-associated FRGs in GC patients are shown in Figure 1(a). Therefore, there were 19 DEGs of them (NOX5, ZFP36, DUSP1, TSC22D3, TXNIP, GABARAPL1, CDO1, TGFBR1, HAMP, NOX4, NNMT, CXCL2, AIFM2, SLC1A4, NF2, SP1, GLS2, MYB, and PSAT1) associated with the prognosis of GC, including 12 upregulated genes and 7 downregulated genes (Figures 1(b) and 1(c)). The PPI network among these genes indicated that DUSP1, NOX4, and SP1 were the hub genes (Figure 1(d)). The correlation between these genes is presented in Figure 1(e).

### 3.2. FRG Clusters Mediated by Prognostic Differentially Expressed Genes

**Figure 2:**
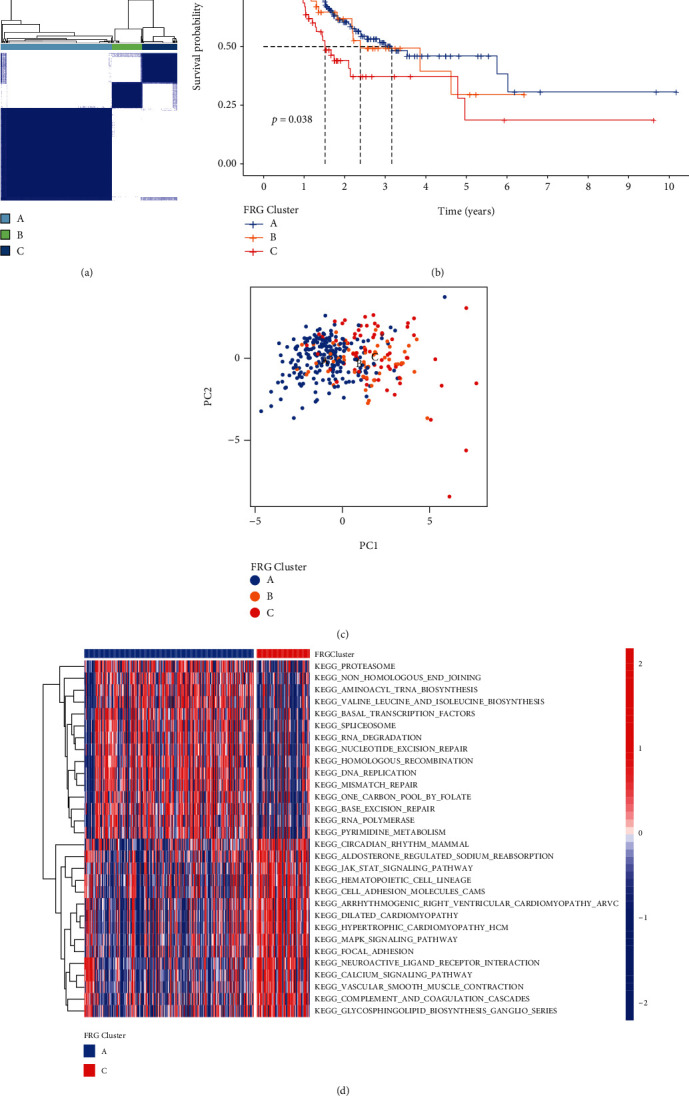
Clusters of FRG expression in GC patients and biological characteristics. (a) Three distinct FRGClusters were identified using unsupervised clustering. (b) Survival analyses for the three FRGClusters based on patients with gastric cancer. (c) Principal component analysis for the transcriptome profiles of three FRGClusters. (d) GSVA enrichment analysis showing the activation states of biological pathways in cluster A vs. cluster C. The heat map was used to visualize these biological processes, and red represented activated pathways and blue represented inhibited pathways.

Three distinct FRG clusters were eventually identified using unsupervised clustering based on the expression of 19 prognostic differentially expressed FRGs. We termed these clusters as FRG cluster A, B, and C, respectively (Figure 2(a)). Cluster A showed particularly prominent survival advantage. As the contrast, cluster C had the worst overall survival (Figure 2(b)). Principal component analysis showing a remarkable difference between cluster A and cluster C (Figure 2(c)).

To explore the biological behaviors among clusters A and C, we performed gene set variation analysis (GSVA). As shown in (Figure 2(d)), cluster A was enriched in DNA replication and transcription regulation of RNA compare with cluster C.

### 3.3. Generation of FRGScore and Its Related Biological Processes in GC

**Figure 3:**
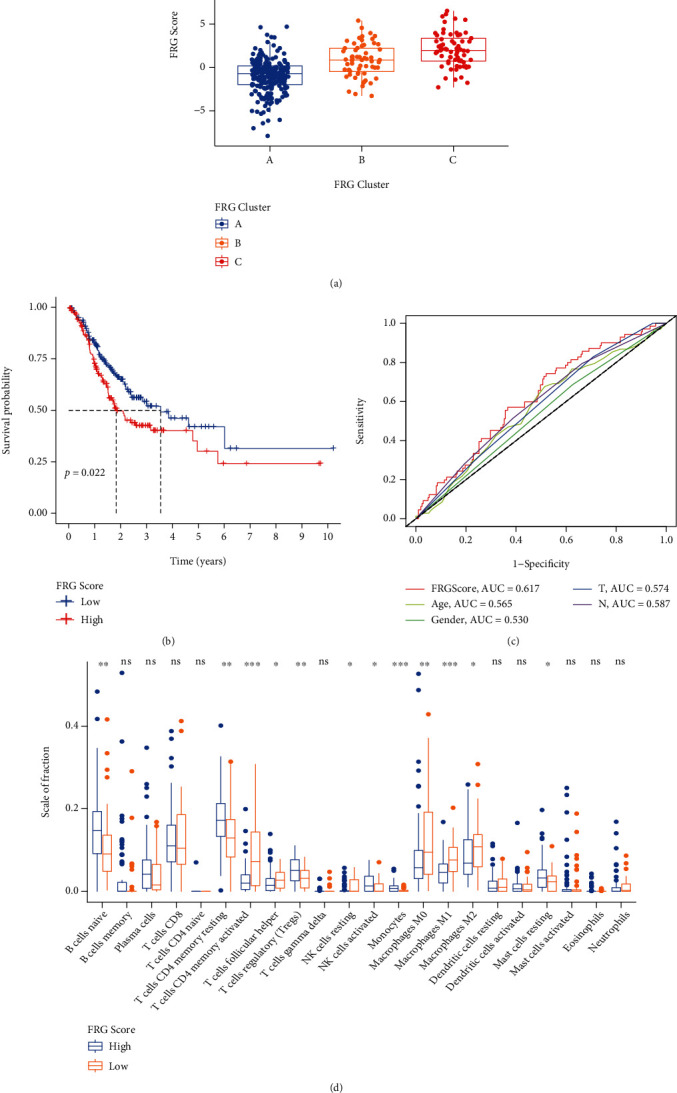
Construction FRGScore. (a) Differences in FRGScore among three FRGClusters. (b) Kaplan-Meier curves indicated FRGScore were markedly related to overall survival of patients in the TCGA cohort. (c) ROC curves of FRGScore and other clinical factors. (d) Different immune cell subset infiltration of low and high FRGScore patient groups.

To study the individual heterogeneity of ferroptosis patterns in GC patients, we constructed FRGScore which showed significant differences between the FRG clusters. The higher FRGScore was obviously concentrated on cluster C and showed a worse survival in GC patients, while the lower FRGScore group was concentrated on cluster A, which was related to the better survival (Figures 3(a) and 3(b)). We used ROC curves to assess the prognostic value of FRGScore and other clinical factors, and among them, FRGScore showed the best prognostic value (Figure 3(c)).

Moreover, the CIBERSORT computational method was used to investigate the association between FRGScore and immune status. The results showed that T cell CD4 memory activation in higher FRGScore patients was lower than those of the lower FRGScore group (Figure 3(d)). These results indicate that FRGScore is related to the immune microenvironment of GC patients.

**Figure 4:**
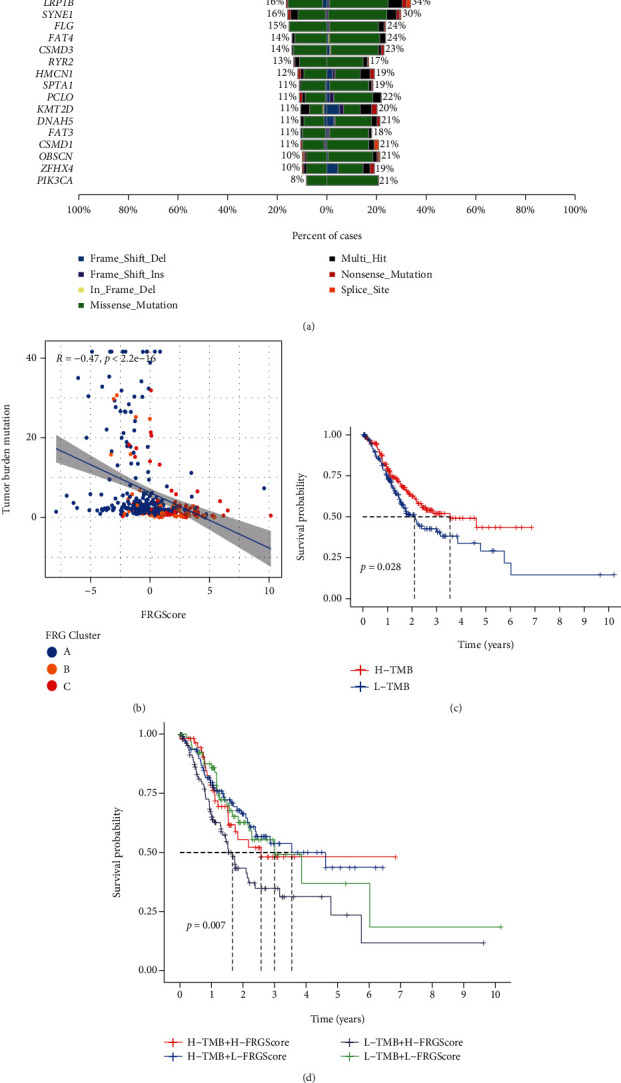
Correlation of FRGScore with TMB. (a) Tumor somatic mutation established by those with high and low FRGScore. (b) Correlations between FRGScore and TMB. (c) Survival analyses for low and high TMB patient groups in the TCGA cohort using Kaplan-Meier curves. (d) Survival analyses for subgroup patients stratified by both FRGScore and TMB using Kaplan-Meier curves.

Then, the distribution differences of somatic mutation between low and high FRGScore groups in the TCGA cohort were analyzed by the maftools package. As shown in Figure 4(a), the low FRGScore group presented more extensive tumor mutation burden than the high score group. The FRGScore and TMB also exhibited a significant negative correlation (Figure 4(b)). Low TMB patients had worse prognosis (Figure 4(c)). We specifically examined the relationship between FRGScore and TMB in prognosis. We found that patients with high FRGScore and low TMB had the worst prognosis (Figure 4(d)). Accumulated evidences indicated that patients with high TMB status presented a durable clinical response to anti-PD-1/PD-L1 immunotherapy. Therefore, these results indirectly demonstrated the differences in tumor FRG patterns could be a crucial factor, which can mediate the clinical response to anti-PD-1/PD-L1 immunotherapy.

### 3.4. FRG RiskScore Is an Independent Biomarker for Prognosis of GC Patients

**Figure 5:**
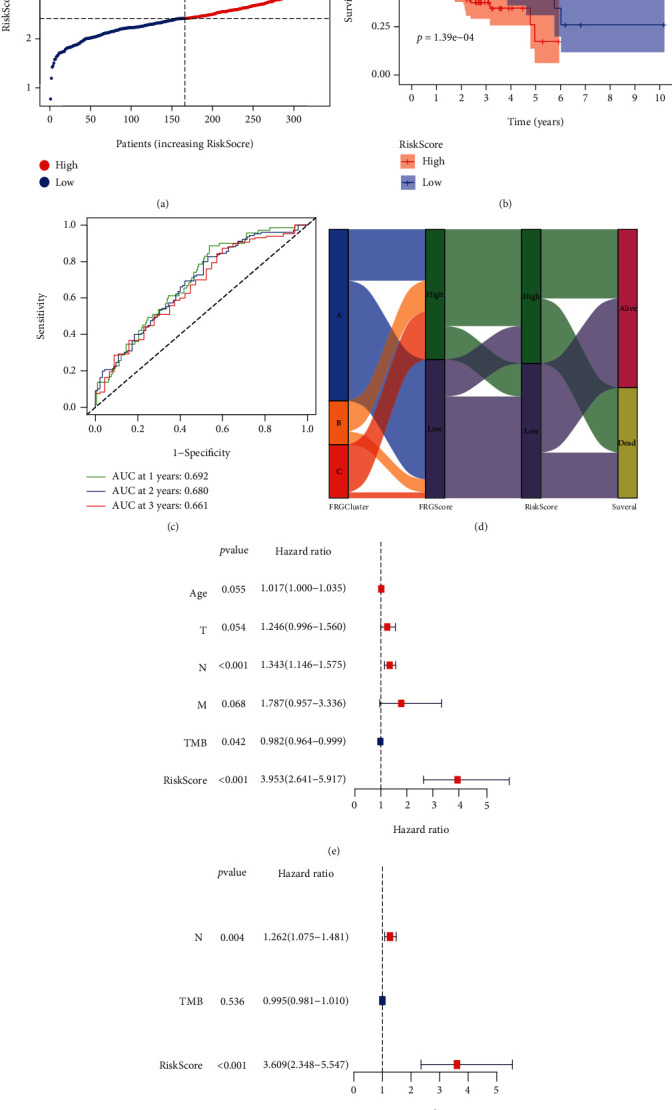
Prognostic analysis of the 13-gene signature model in the TCGA cohort. (a) The distribution and median value of the RiskScores in the TCGA cohort. (b) Kaplan-Meier curves for the OS of patients in the high-risk group and low-risk group in the TCGA cohort. (c) AUC of time-dependent ROC curves verified the prognostic performance of the RiskScore in the TCGA cohort. (d) Alluvial diagram showing the changes of FRGClusters, FRGScore, RiskScore, and survival state. (e, f) Results of the univariate (e) and multivariate (f) Cox regression analyses regarding OS in the TCGA derivation cohort.

The expression profile of the 19 FRGs was used to establish the risk scoring system using LASSO Cox regression analysis, and 13 genes were identified. The RiskScore was calculated as follows: RiskScore = SUM (0.221∗expression level of NOX4 + 0.882∗expression level of NOX5 − 0.174∗expression level of GLS2 − 0.004∗expression level of MYB + 0.002∗expression level of TGFBR1 − 0.058∗expression level of NF2 − 0.027∗expression level of AIFM2 + 0.001∗expression level of ZFP36 − 0.021∗expression level of SLC1A4 + 0.001∗expression level of TXNIP − 0.007∗expression level of CXCL2 + 0.001∗expression level of HAMP − 0.017∗expression level of SP1 (Figure 5(a)). The Kaplan-Meier curve revealed that the prognosis of low-risk patients was significantly better than that of the high-risk group (Figure 5(b)), suggesting great sensitivity and specificity of the prognostic signature in predicting OS. Time-dependent ROC curves was performed to evaluate the predictive performance of RiskScore for overall survival outcome. The area under the curve (AUC) reached 0.692 at 1 year, 0.680 at 2 years, and 0.661 at 3 years, which shows a high accuracy (Figure 5(c)). To study the relationship of patients in different ferroptosis assessment systems, the alluvial diagram was constructed to showing the variation of FRGClusters, FRGScore, RiskScore, and survival state (Figure 5(d)). Univariate and multivariate Cox regression analyses were then performed to determine whether RiskScore was a predictor for OS independent of other clinical features. We found that *N* stage (HR = 1.262) and RiskScore (HR = 3.609) were independent predictors for OS (Figures 5(e) and 5(f)).

### 3.5. RiskScore Is a Biomarker for Immune Checkpoint Therapy in GC Patients

**Figure 6:**
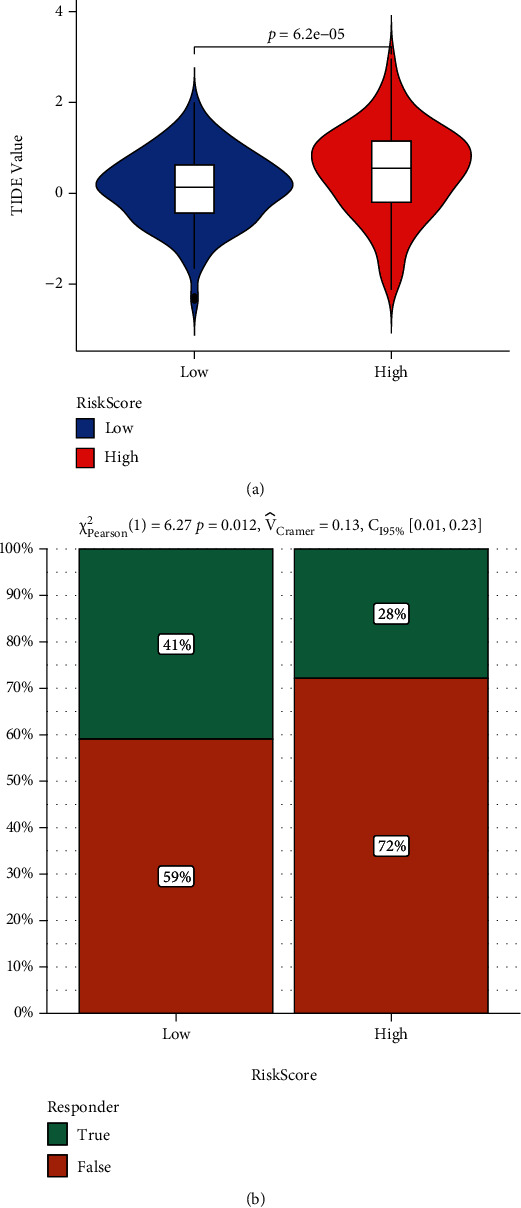
Predictions of the immunotherapy response in GC patients. (a) The violin plots present of immune dysfunction in high and low RiskScore groups. (b) The likelihood of the clinical response to antiPD1 and anti-CTLA4 therapy for high and low RiskScore patients from the TCGA cohorts. True represents immunotherapy responders, while false represents immunotherapy nonresponders.

Afterwards, the tumor immune dysfunction and exclusion (TIDE) algorithm analysis was used to predict the immune checkpoint therapy response based on RiskScore in GC patients. Interestingly, according to the results shown in Figures 6(a) and 6(b), GC patients with lower RiskScore had less immune dysfunction and were more likely to respond to immunotherapy, suggesting that RiskScore can be used as reference index for clinical treatment of gastric cancer patients, as whether use immunotherapy for these patients.

### 3.6. Construction and Validation of the Predictive Nomogram in GC

**Figure 7:**
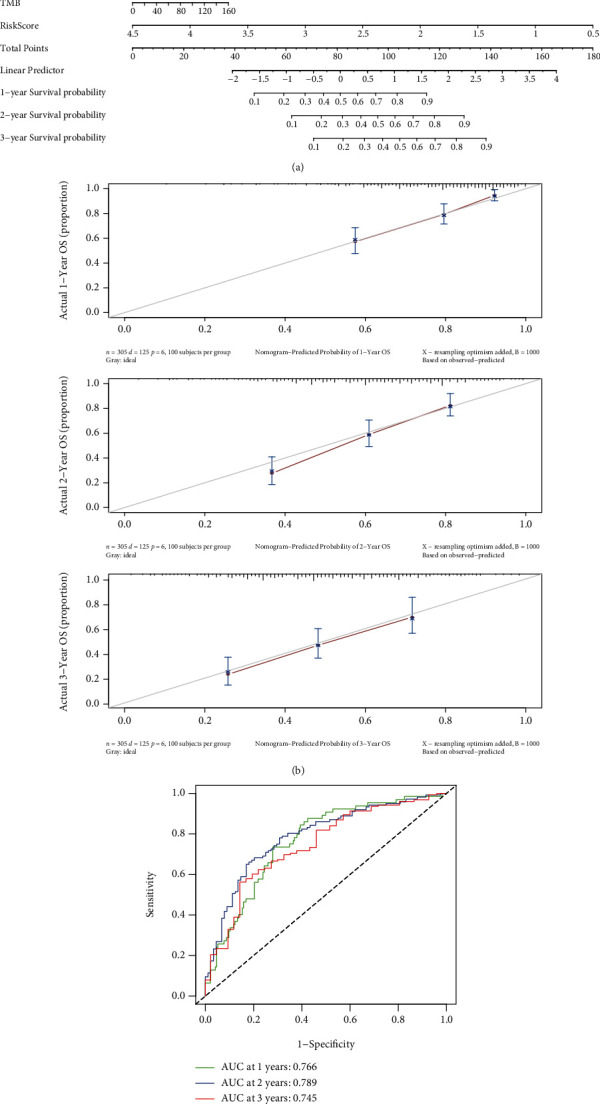
Construction of a predictive nomogram. (a) The nomogram for predicting the OS of patients with GC at 1, 2, and 3 years. (b) Calibration curves of the nomogram for OS prediction at 1, 2, and 3 years. (c) ROC curves to evaluate the predictive ability of the nomogram.

A nomogram was developed to quantify the prediction of individual survival probability for 1, 2, and 3 years (Figure 7(a)). The C-index of the nomogram was 0.72 (95% CI: 0.67–0.76). The calibration curves indicated great consistency between predicted OS and actual observation at 1, 2, and 3 years (Figure 7(b)). Then, ROC curves were generated to verify the predictive value of the nomogram. The AUCs for 1-, 2-, and 3-year OS were 0.766, 0.789, and 0.745, respectively, in the TCGA database (Figure 7(c)).

### 3.7. Construction of the ceRNA Network

**Figure 8:**
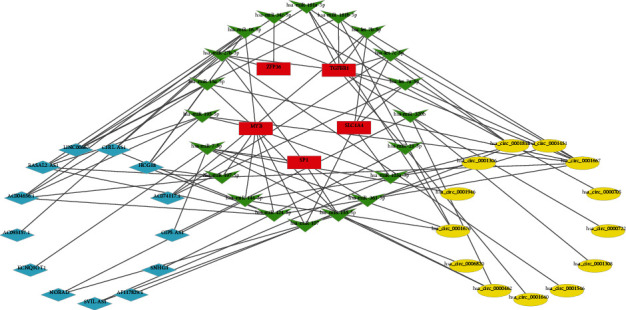
Construction of the circRNA–miRNA–lncRNA–mRNA regulatory network.

To inquiry about the regulatory network of FRGs in gastric carcinoma, we used GDCRNATools to find 38 miRNA target 6 FRGs in the prognosis model, and 23 lncRNAs bind to the miRNAs. Then, we find 13 different expressed circRNAs which had a spongy effect to the miRNAs in starBase. Finally, we constructed the circRNA–miRNA–lncRNA–mRNA regulatory network based on 15 circRNA, 20 miRNA, 13 lncRNA, and mRNA (Figure 8). This is a kind of complicated ceRNA network rather than single RNA molecule [11], which can induce the relative phenotype of malignant tumor such as gastric cancer and colorectal cancer, including proliferation, inhibition, indeterminate growth, inducing angiogenesis, and immune escape [12–16]. Therefore, constructing this ceRNA network can provide an essential reference to the process of relative malignant tumor including gastric cancer and it may provide a novel diagnosis biomarker and immunotherapy biomarker for the therapy of associated cancer in clinic.

## 4. Discussion

Gastric tumorigenesis is a multifactorial, multicellular, and multistep process. Helicobacter pylori infection, environmental factors (salted food intake, alcohol consumption, and low socioeconomic status), and population-specific genetic risk factors can induce the occurrence of GC. However, the detailed mechanism for GC still remains not clear. Nowadays, the major GC diagnosis methods include imaging examination, serum tumor marker examination, and tissue biopsy. But as a gold standard method, tissue biopsy can cause obvious surgical trauma and hysteresis. In addition, many of the GC patients are diagnosed in the later stage with worse prognosis because of the lack of specific symptoms of early stage in GC. What is more, the main available treatments, including radiotherapy and chemotherapy, have intense side effects on patients. Sometimes, the effect of chemotherapy is obvious, but it cannot be sustained for a long time due to drug resistance. In that case, it is necessary to learn more practical and effective methods to improve GC diagnosis and treatment, and indispensable to figure out key molecular markers that can predict the prognosis of GC.

Although in the past few years, the mechanism of tumor susceptibility to ferroptosis disorder has been the focus of research, the potential regulatory role between tumor immunity and ferroptosis disorder remains unclear. Here, we revealed three distinct ferroptosis clusters in GC patients based on 19 prognostic differentially expressed FRGs. These three patterns had significantly distinct overall survival. Cluster A was characterized by the activation of DNA replication and transcription regulation of RNA. Since most patients in cluster A had lower FRGScore and FRGScore was negatively correlated with TMB, the active DNA replication in cluster A may be related to the TMB. Some evidences demonstrated patients with high TMB status can present a durable clinical response to anti-PD-1/PD-L1 immunotherapy. The programmed cell death protein 1 (PD-1) pathway can elicit the immune checkpoint response of T cells, causing tumor cells capable to evade immune surveillance and become highly refractory to conventional chemotherapy [17]. In our study, CD4+ memory T cells in the lower FRGScore group were more active, and CD4+ helper T cells provide an opportunity to enhance T cell response to tumor-associated antigens without deleterious autoimmunity [18]. In that case, these results indirectly demonstrated that the ferroptosis patterns of gastric cancer could be a crucial factor mediating the clinical response to anti-PD-1/PD-L1 immunotherapy.

Our research demonstrated that low ferroptosis status is significantly associated with better outcomes from different perspectives. And compared to other clinical features, FRGScore had the best prognostic value. Meanwhile, we found patients with high FRGScore and low TMB had the worst prognosis. In this research, transcriptomic data and the relative clinical information were used to identify key ferroptosis-related genes, which are significantly valuable in GC prognostic prediction. Then, we constructed a survival model with superior accuracy to predict the prognosis for GC patients through these ferroptosis-related genes. Through analysis, it can be evaluated that this model is effective, independent, and robust with high reliability. As far as we know, this is the first report focusing on the relationship between iron deposition-related gene markers and prognosis related to the prognosis of gastric cancer patients. The prognostic model constructed in the present study was composed of 13 FRGs (NOX4, NOX5, GLS2, MYB, TGFBR1, NF2, AIFM2, ZFP36, SLC1A4, TXNIP, CXCL2, HAMP, and SP1). A previous study had reported that erastin-induced accumulation of lipid ROS is abolished by NOX4 inhibitor [19]. NOX4 inhibition can reduce the cystine deprivation-induced cell death and lipid ROS, indicating its essential role in ferroptosis [20]. Moreover, NOX5 is also a key regulator of ferroptosis. Enhancing the NOX5 activity on cell membrane cause subsequently concentrates the local ROS oxidization and activates oncoprotein-Src to promote malignancy of tumor cells [21]. The high expression of NOX5 mRNA indicated a poor survival outcome in stage III/IV GC patients, but not in stage I/II GC patients. These observations indicated that NOX5 may be an unfavorable prognosis indicator for late-stage GC patients [22]. As a key enzyme for glutamine metabolism, GLS2 can regulate the biosynthesis of GSH during the ferroptosis process and serve as a target of the p53 gene [23]. Physcion 8-O-*β*-glucopyranoside (PG) significantly trigged the GC cell ferroptosis and suppressed biological behavior through downregulating the inhibitory effect of miR-103a-3p on GLS2 expression and promoted ROS level and MDA generation, but the prognosis role of GLS2 in GC is still unclear [24]. Our study showed that high GLS2 expression is a potential biomarker for better prognosis of GC. As a tumor suppressor gene, NF2 can be activated by E-cadherin and inhibits ferroptosis in endothelial cells [25]. SLC1A4, a Na-dependent neutral amino acid transporter, is considered to take part in the various pathobiological processes, including tumorigenesis. But its role in GC is still not clear enough [26]. ZFP36 overexpression can trigger autophagy inactivation, block autophagic ferritin degradation, and eventually confer resistance to ferroptosis [27]. TXNIP played a role as a potent negative regulator for glucose uptake and aerobic glycolysis. Therefore, the aerobic glycolysis will be inhibited and substrate flux will be decreased through the pentose phosphate pathway to produce less NADPH and GSH because of the reduced TXNIP, which results in trigging ferroptosis [28]. Ferroportin (Fpn) worked as a negative regulator of ferroptosis through reducing intracellular iron concentration, and hepcidin (HAMP) could prevent erastin-induced ferroptosis by degrading Fpn [29]. At last, such as SP1, ASCT1, and CXCL2, these genes also play crucial roles in ferroptosis, but most of these genes are still not clear in the regulation and mechanism of GC progression. Thus, the immune processes can contribute to GC development and prognosis, which can be proved by the involvement of all the above-mentioned ferroptosis-related genes, immune cell infiltration, and related pathways. Therefore, the proposed model can identify novel biomarkers for further research. And we also find that the low-risk group is more sensitive to immunotherapy while the high-risk group is less sensitive, which may give the potent reference to clinical treatment.

For the ceRNA network analysis, five of the ferroptosis-related genes, ZFP36, TGFBR1, MYB, SP1, and SLC1A4, are associated with ceRNA process. Some researches have already demonstrated that circNRIP1 sponges miR-149-5p can regulate the expression level of AKT1 and act as a tumor promoter in GC [30]. Moreover, Ren et al. found that ILF3-AS1 can enhance the expression of PTBP3 as an miR-29a sponge to promote the proliferation and metastasis of GC cells [31]. However, more and more researches need to be done to further study the concrete process. Learning the relationship between GC and relative ceRNA network helps us understand the concrete mechanisms of development in GC with different degrees of immune cell infiltration [32].

## 5. Conclusion

In summary, we had constructed a ferroptosis-related gene signature model to predict GC prognosis with high accuracy, which may provide a novel prognostic model in clinic. The identification of ferroptosis-related genes may provide new potential biomarkers for research on the molecular mechanisms and personalized treatment decisions for patients with GC. The sensitivity of immunotherapy for GC is various in these two groups, and the low-risk group shows a higher sensitivity, which may provide references to clinic treatment. Moreover, ceRNA is also strongly associated with some ferroptosis-related gene signatures. However, more researches need to be done for further study.

## Data Availability

The data and materials used to support the findings of this study are available from the corresponding author upon request.
